# Two Distinct Faces of Vitamin C: AA vs. DHA

**DOI:** 10.3390/antiox10020215

**Published:** 2021-02-01

**Authors:** Luciano Ferrada, Rocío Magdalena, María Jose Barahona, Eder Ramírez, Cristian Sanzana, José Gutiérrez, Francisco Nualart

**Affiliations:** 1 Center for Advanced Microscopy CMA BIO BIO, Faculty of Biological Sciences University of Concepcion, Concepción 4030000, Chile; luferrada@udec.cl (L.F.); mjbarahona@cmabiobio.cl (M.J.B.); 2 Laboratory of Neurobiology and Stem Cells NeuroCellT, Department of Cellular Biology, Faculty of Biological Sciences, University of Concepcion, Concepción 4030000, Chile; rmagdalena@udec.cl (R.M.); ramirez.ed27@gmail.com (E.R.); csansana@udec.cl (C.S.); 3 Laboratory of Transcriptional Regulation, Department of Biochemistry and Molecular Biology, Faculty of Biological Sciences, University of Concepcion, Concepción 4030000, Chile; lgutier@udec.cl

**Keywords:** vitamin C, signal transduction, cancer, cell death, necroptosis

## Abstract

Historically, vitamin C has been associated with many regulatory processes that involve specific signaling pathways. Among the most studied signaling pathways are those involved in the regulation of aging, differentiation, neurotransmission, proliferation, and cell death processes in cancer. This wide variety of regulatory effects is due to the fact that vitamin C has a dual mechanism of action. On the one hand, it regulates the expression of genes associated with proliferation (Ccnf and Ccnb1), differentiation (Sox-2 and Oct-4), and cell death (RIPK1 and Bcl-2). At the same time, vitamin C can act as a regulator of kinases, such as MAPK and p38, or by controlling the activation of the NF-kB pathway, generating chronic responses related to changes in gene expression or acute responses associated with the regulation of signal transduction processes. To date, data from the literature show a permanent increase in processes regulated by vitamin C. In this review, we critically examine how vitamin C regulates these different cellular programs in normal and tumor cells.

## 1. Introduction

The reduced form of vitamin C (ascorbic acid, AA) is an essential micronutrient of small size; it is soluble in water and has two dissociable protons with pKa values of 4.2 and 11.8. At physiological pH, its reduced form predominates as the monovalent ascorbate anion (AA); when it loses the second proton, it is oxidized to dehydroascorbic acid (DHA) [1,2,3]. Most mammals can synthesize vitamin C from D-glucose in the liver, except guinea pigs, bats, and higher primates, including humans, due to the absence of the enzyme L-gulonolactone oxidase, which catalyzes the last step of the biosynthesis of vitamin C [4]. Therefore, to meet the body’s requirements, vitamin C must be incorporated into the diet [1]. The best-known function of vitamin C is as an antioxidant agent that can act as a cofactor of enzymatic reactions involved in the synthesis of catecholamines, carnitine, cholesterol, amino acids, and some hormonal peptides, as well as in the maintenance of brain function and the protection of central nervous system (CNS) structures [1,3,5,6,7].

AA uptake in different cells is performed by the sodium-ascorbate cotransporters SVCT1 and SVCT2, which stereospecifically transport the reduced form of vitamin C, L-ascorbate [8,9,10,11]. Vitamin C can also be transported in its oxidized form, DHA, through the facilitative glucose transporters GLUT1, GLUT2, GLUT3, GLUT4, and GLUT8 [12,13,14,15,16]. However, for a long time, it has been postulated that the contribution of DHA to the accumulation of vitamin C in tissues is relatively low [3,17,18,19]. 

All of the aforementioned functions of vitamin C can be considered classical because they have been widely described and characterized in the literature. Thus, in this review, we will focus on describing the new and emerging functions for vitamin C in specific tissues, including the regulation of various signaling pathways that control proliferation and cell death processes in cancer. Furthermore, we will discuss how many of the functions associated with vitamin C could be related to its oxidized form, DHA, a possible master regulator of the processes associated with kinase activity, proliferation, and cell death.

## 2. Molecular Pathways Regulated by Vitamin C

**Figure 1:**
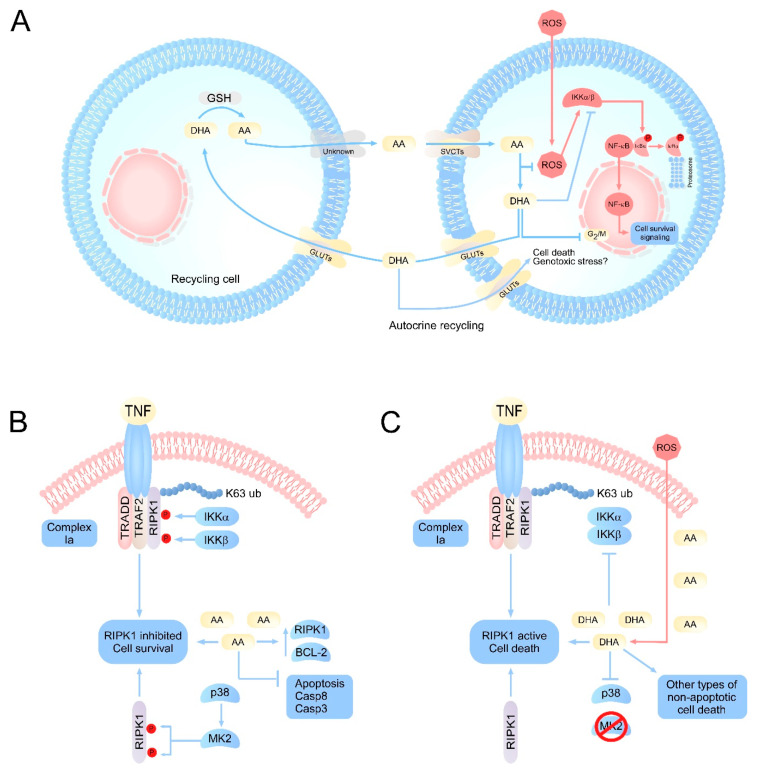
Integrative vision of the principal molecular pathways regulated by vitamin C. (**A**) Scheme of vitamin C recycling in normal cells or cells with oxidative stress. Under normal conditions, AA concentrations remain homeostatically stable due to the efficient recycling of DHA by specialized cells. However, under conditions of oxidative stress or inefficient recycling, an accumulation of intracellular DHA can occur. DHA would target the inhibition of IKK α/β, metabolic enzymes such as GAPDH, as well as the production of genotoxic stress, resulting in the induction of cell death. (**B**) Intracellular effects of vitamin C on signaling pathways associated with cell death. The physiological levels of AA would have a protective function intracellularly, favoring the inhibition of apoptosis by inducing overexpression of antiapoptotic genes, as well as caspases. At the same time, AA could maintain RIPK1 in its inhibited state, which favors cell survival. (**C**) Under pathophysiological or acute oxidative stress conditions, ROS overload induces a massive oxidation of AA to DHA, intracellularly. The accumulation of DHA results in the inhibition of IKK α/β, and p38, which can trigger the activation of RIPK1 and cell death due to necroptosis, in cells that accumulate high concentrations of vitamin C, such as neurons. AA: ascorbic acid; DHA: dehydroascorbic acid; SVCT2s: sodium-dependent vitamin C transporter; GLUTs: glucose transporters; RIPK1: receptor-interacting serine/threonine-protein kinase 1; MK2: p38MAPK-activated protein kinase 2; TRADD: TNFR1-associated death domain protein; TRAF2: TNF receptor associated factor 2.

One of the first targets for vitamin C was discovered via its relationship to the NF‒κB pathway. In this pathway, vitamin C has an inhibitory function; in studies carried out in endothelial cells, millimolar doses of AA inhibited NF-κB and IL-8 activation in response to tumor necrosis factor (TNF) [20]. In this study, the authors also evaluated the toxicity generated by high doses of vitamin C supplementation and did not detect cell damage or lipid peroxidation [20,21]. Furthermore, they were able to determine that the inhibition of the NF-κB pathway was not due to the antioxidant activity of vitamin C, but rather to the direct inhibition of IκB kinase α/β (IKKα/β) [20,21,22,23]. In line with this notion, IKKα/β is a kinase responsible for the phosphorylation of IκBα protein that maintains NF-κB-p65 in the cytoplasm [24]. IκBα phosphorylation is a signal for proteasomal degradation of this protein, allowing NF-κB-p65 nuclear translocation (Figure 1A), triggering the activation of specific genes [24,25,26]. In line with these findings, it was postulated that AA is a regulator of IKKα/β activity; however, subsequent studies determined that AA has no action on IKKα/β [22,27]. Interestingly, it was shown that DHA was a regulator of IKKα/β mediated by directly binding to this kinase, inhibiting it, and finally controlling the activity of NF-κB [27]. This function of DHA was determined through immunoprecipitation experiments using p-IκBα-GST where derivatives of vitamin C, AA, DHA, oxalic acid, and threonic acid were used. Only treatment with DHA inhibited IκBα-phosphorylation, and this inhibition was mediated by DHA directly blocking the activity of IKKα/β and p38, likely competing for the binding of ATP to the active site of IKKβ [22,27]. Given this evidence, it was concluded that vitamin C has a dual action against reactive oxygen species (ROS). Intracellularly, AA would fulfill its antioxidant function by neutralizing ROS, generating DHA. Thus, the intracellular accumulation of DHA would block the activation of NF-κB, involving vitamin C in signaling processes that control inflammatory responses and cell death among others (Figure 1A).

Another kinase-dependent pathway that is regulated by vitamin C is that of the mitogen-activated protein kinases (MAPK), which involves three other MAPK-dependent pathways, extracellular signal-regulated kinases (ERK), c-Jun N-terminal kinases (JNK), and p38 kinase, which are involved in proliferation, differentiation, and apoptosis, respectively [10,28,29]. The first studies examining the relationship between vitamin C and MAPK concluded that vitamin C was involved in in vitro cell death processes, but the mechanism of action was unknown. Thus, the possible regulation of MAPK-ERK mediated by vitamin C was analyzed. For this, leukemia cell lines were treated with AA (0-500 µM) for 1 to 3 h in order to analyze ERK activation by in vitro phosphorylation assays. In cells treated with concentrations as low as 100 μM AA, phosphorylation of ERK and therefore activation was induced [10,30]. Thus, it was proposed that the regulation of ERK mediated by vitamin C would be associated with eventual apoptotic processes that are observed in certain tumor lines when treated with vitamin C because ERK activation is associated with proliferative processes and cell death. However, to date, it has been shown that the use of pharmacological doses of AA induces tumor death from conventional necrosis due to the extracellular generation of H_2_O_2_ [31,32], as discussed in detail later. At the same time, it has also been shown that AA can antagonize apoptosis in cancer cells induced by classical mechanisms, such as treatment with doxorubidicin, TRAIL, or FAS [33,34,35]. In line with this notion, treatment with physiological doses of AA in neuronal cultures induces overexpression of antiapoptotic genes, such as Bcl-2, and decreases the expression of proapoptotic genes, such as Bax and caspase 8 [19]. Thus, the current evidence suggests that physiological doses of AA could inhibit apoptosis rather than activate this death pathway. Furthermore, AA-mediated ERK activation could be associated with neuronal arborization mechanisms, which would be an indicator of neuronal “good health” [10]. 

Subsequent studies have shown that vitamin C has a regulatory role on MAPK. Specifically, treatment with AA 60 μM for short periods of time (15–20 min) induces epithelial cell proliferation that is dependent on the activation of ERK, but not p38 [36]. However, regulation of AA on MAPK seems to be dependent on its concentration and cell type; in melanoma cells treated with concentrations of 50–500 μM AA, p38 phosphorylation increased, but no increase in ERK phosphorylation was observed [28]. Furthermore, treatment with 0.5–1 mM AA could have an inhibitory effect on MAPK [37,38]. These data strongly suggest that there is a delicate balance between the dose of AA and the molecular target that is affected by the treatment. Given the accumulating evidence, it is tempting to speculate that, at low doses of AA, MAPK activation is favored, while at high doses, this pathway could be inhibited. Some of the main effects of vitamin C and the cell models used are summarized in Table 1. Thus, the role of vitamin C in the regulation of MAPK is unclear, because the literature has not been able to determine its specific role in this signaling pathway. This makes it difficult to propose a possible treatment against a pathology, where a therapy that includes pharmacological inhibitors or regulators of MAPK is used in conjunction with AA. What is clear, to date, is that AA is either a positive or negative regulator of MAPK not DHA, given that all experiments were carried out over short periods of time and under conditions where the oxidation of AA is not favored; thus, DHA apparently would not be involved in the regulation of MAPK/ERK.

## 3. Vitamin C as a Cell Cycle Regulator

The cell cycle is regulated by interactions between cyclins and cyclin-dependent kinases (cdk). The cyclin‒cdk complex is up- or downregulated by phosphorylation. When DNA damage occurs, cells can be arrested at the G1/S, S, or G2/M cell cycle checkpoints for DNA repair or to enter cell death processes [44,45]. In addition, AA has the ability to regulate the cell cycle directly. During periods of oxidative stress, AA can trigger cell cycle arrest at the S-phase checkpoint [40]. In line with this notion, a recent study in primary human fibroblasts showed that AA treatment decreased the expression of 31 genes [46]. Interestingly, of these 31 genes, 12 corresponded to tRNA synthetases and translation initiation factor subunits, which are required for cell cycle progression [46]. Strikingly, the effects on the cell cycle generated by vitamin C are frequently observed when it is used in combination with pro-oxidant molecules [47,48,49,50]. When AA was used in combination with agents that induce oxidative stress, growth was inhibited, and the cell cycle arrested at the G2/M checkpoint [41]. Co-incubation of AA together with pro-oxidant molecules triggered cell death, possibly due to necrosis [48]. This suggests that the effects observed and attributed to AA could possibly be triggered by DHA because, when AA is co-incubated with pro-oxidant molecules, it must neutralize ROS and oxidize to DHA. In addition, the treatments were generally for long periods of time and involved a single high-concentration supplementation, which would favor the oxidation of AA. Currently, there is evidence that supports the hypothesis that DHA could be the trigger for cell cycle arrest; when primary hepatocyte cultures were treated with AA, DNA synthesis and cell proliferation were observed [51]. However, when cells were treated with DHA, some proliferation was induced, but it was not sustained [51]. Thus, it is again unclear whether the impact of vitamin C on cell proliferation is due to AA or DHA. Thus, determining which form of vitamin C controls the regulatory functions of proliferation is essential before possible pharmacological use, such as its use as an antineoplastic.

## 4. Vitamin C as an Enzymatic Cofactor

Epigenetic modifications are reversible changes that affect the genomic structure of DNA, which dictates the accessibility of transcriptional machinery to its sequence, thus regulating gene expression [52]. In particular, chromatin modifications include DNA methylation/demethylation and histone modification, which are introduced by the action of different enzymes. In this context, the influence of various metabolites on enzymatic activity has been widely described [53]. For example, different intermediaries of glycolysis and the citric acid cycle can introduce modifications, such as acetylation or methylation [53]. In the same way, the action of various vitamins in the generation of epigenetic modifications has also been reported [54], indicating that these molecules would also play a role in enzymatic function. Particularly, vitamin C can act as a cofactor of the Fe^2+^ and 2-oxoglutarate (2-OG) family of dioxygenase enzymes [55], which includes important epigenetic regulators, such as Jumonji-C domain-containing histone demethylases (JHDMs) and TET hydroxylases, the latter associated with the conversion of 5-methylcytosine (5mC) into 5hydroxymethylcytosine (5hmC) in DNA [56]. 

Vitamin C deficiency generates scurvy, a condition in which collagen synthesis is mainly affected; it acts as a cofactor for proline hydroxylase and lysine hydroxylase [57], which are part of the Fe^2+^ and 2-OG-dependent dioxygenase family. Although they have various substrates, they share a conserved mechanism of action: a Fe^2+^ ion is coordinated at the enzyme’s active site, which binds to 2-OG, permitting entry of the substrate and binding of an oxygen molecule [55]. Subsequently, the oxidative decarboxylation of 2-OG and the generation of ROS will oxidize the substrate and release secondary products [58]. In this context, vitamin C would be essential for maximum enzymatic activity, and it has been reported that the need for vitamin C in these reactions arises from its function as an electron donor to maintain an iron pool in its oxidation state +2 [59,60]. However, previous reports show that the effect of vitamin C is not reproduced in the presence of other antioxidant agents, and its direct interaction with the catalytic domain of TET enzymes has been described, which would indicate a specific role of vitamin C in the dioxygenase family [61]. Nonetheless, it should be noted that vitamin C has been associated with iron metabolism, increasing its uptake into the intracellular environment (and thus increasing the labile iron pool of Fe^2+^) [62]; therefore, vitamin C may be necessary for the maintenance of adequate levels of Fe^2+^. 

Regarding the role of vitamin C in the action of epigenetic enzymes, one of the first analyses was performed on methylated oligonucleosomes in vitro, which were subjected to a histone demethylation reaction in the presence of a nuclear pellet obtained from HeLa cells. In these cells, the production of formaldehyde decreased by ~50% in the absence of vitamin C, indicating that it would be necessary for histone demethylase activity [63]. On the other hand, in vitro DNA demethylation assays in the presence of the catalytic domain of TET2 and different concentrations of vitamin C showed a progressive increase in 5hmC levels in relation to control conditions, an effect that was observed in a time-dependent manner, suggesting that vitamin C accelerates the hydroxymethylation reaction [64]. Additionally, the incubation of vitamin C with the catalytic domain of TET2 showed a progressive extinction of the latter’s intrinsic fluorescence (determined by the presence of tyrosine residues that intrinsically fluoresce) in a concentration-dependent manner, thus suggesting a direct interaction between these two molecules [64]. 

## 5. Cellular Reprogramming Mediated by Vitamin C

A role for vitamin C has also been described in cellular reprogramming, a process in which somatic cells are reprogrammed into an undifferentiated state after the introduction of transcription factors associated with the regulation of pluripotency, thus generating induced pluripotent stem cells (iPSCs). An assay performed on mouse embryonic fibroblasts (MEFs) transduced with these transcription factors (*Sox-2*, *Oct-4*, *Klf4*, *c-Myc*) showed that the addition of vitamin C to the culture medium favors cell reprogramming, an effect that is not reproduced by other antioxidant agents, such as vitamin B1, reduced glutathione, or sodium selenite, and thus would not be associated with the antioxidant function of vitamin C [65]. 

Subsequent studies showed that, during reprogramming, vitamin C lowers H3K36me2/me3 levels or histone marks associated with transcriptional activation, an effect that would be mediated by the action of vitamin C-dependent histone demethylases, JHDM1a and 1b [66]. In particular, JHDM1b has been associated with cell proliferation; thus, overexpression in MEFs reprogrammed in the presence of vitamin C significantly increased the proliferation and expression of various cell cycle regulatory genes, such as *Ccnf, Ccnb1*, and *AurkB* [66]. Additionally, treatment with JHDM1b and vitamin C during reprogramming significantly decreased H3K36me2 levels, concomitant with an enrichment of repressive histone mark H3K27me3 in the *Ink4/Arf* locus, which encodes different tumor suppressor proteins that induce cellular senescence [67] and whose expression decreases when overexpressed JHDM1b and vitamin C are present [66]. Taken together, these studies indicate that vitamin C favors cellular reprogramming, at least in part by delaying cell senescence in a process that would involve its action on histone demethylase, JHDM1b. 

In 2017, a study was published in which high levels of vitamin C were observed in human and mouse hematopoietic stem cells (HSCs) in relation to other, more differentiated cell populations [68]. Vitamin C-deficient mice (*Gulo^−/−^*) have a higher frequency of HSCs and lower genomic levels of 5hmC, effects that reproduce what has been observed in the presence of mutations that inactivate *Tet2*, which increase HSC proliferation and are prevalent in leukemia development [68]. 

A very common mutation in the development of acute myeloid leukemia corresponds to an internal tandem duplication of Flt3 (*Flt3^ITD^*), a tyrosine kinase receptor associated with the proliferation and maturation of HSCs, which generates a gain in its function [69]. Transplant of mutant cells into *Gulo^−/−^* mice increased proliferation of myeloid lineage cells in relation to control animals, thus indicating that vitamin C deficiency cooperates with this mutation to promote myelopoiesis, a process that would be mediated mainly by TET2 [68]. Additionally, *Gulo^−/−^* mice transplanted with mutant cells, which are deficient in *Tet2*, showed an increase in the percentage of myeloblasts in blood and the development of a series of myeloproliferative pathologies, which can be reversed with vitamin C supplementation [68]. In this way, vitamin C deficiency, in combination with other mutations, such as those that inactivate *Tet2*, can favor the development of myeloproliferative pathologies. In fact, vitamin C treatment of *Tet2*-defficient hematopoietic stem progenitor cells (HSPCs) mimics Tet2 restoration, increasing 5hmC levels and blocking aberrant self-renewal capacity [70]. Furthermore, AA treatment for 24 weeks lowers white blood cell count and hinders disease progression in vivo [70], thus highlighting the importance of adequate serum vitamin C levels. 

## 6. Intracellular Localization of SVCT2 and Mitochondrial Function

To date, the entry of vitamin C into the mitochondria has been a subject of debate. It is postulated that vitamin C enters the mitochondria through GLUTs [71] or SVCT2 [72]. However, the entry of AA into the mitochondria through SVCT2 would be limited to certain tissues [72]. At the same time, AA uptake through SVCT2 in the mitochondria is highly disadvantaged by the low concentration of intracellular Na^+^ available [73]. On the contrary, the incorporation of DHA into the mitochondria through GLUTs does not have the limitations of ion availability and occurs by facilitated diffusion as a universal mitochondrial vitamin C accumulation mechanism [73]. Moreover, our super-resolution data show that SVCT2 does not colocalize with mitochondria, at least in rat neuronal primary cultures or neuroblastoma models [19], suggesting that, at least in the brain, vitamin C could be incorporated by the mitochondria through GLUTs in the form of DHA. Interestingly, when neuronal cells are previously loaded with AA and subsequently subjected to oxidative stress, massive mitochondrial fragmentation and neuronal death are observed [19]. We hypothesize that mitochondrial fragmentation could be the consequence of a massive intracellular production of DHA, which would favor the metabolic alterations induced by this molecule. Using genetic editing through CRISPR/Cas9, we deleted SVCT2 in mouse neurons [19] and found that the effect of cell death induced by intracellular oxidation of vitamin C is lost, which could be due to the inability to transport AA. Unexpectedly, SVCT2-null neurons were 10-fold more sensitive to H_2_O_2_-induced cell death than wild-type neurons in the absence of vitamin C [19]. Similarly, SVCT2 knockdown using shRNA decreased hepatocellular carcinoma tumor size and increased the toxicity of antineoplastic drugs, such as cisplatin and sorafenib [74]. These observations led us to propose that SVCT2 could have other functions beyond transporting AA that are involved in survival mechanisms or resistance to oxidative stress. Interestingly, in breast cancer cells, an opposite effect has been observed, where an increase in SVCT2 levels would favor AA-mediated toxicity, while the use of siRNA against SVCT2 confers resistance to AA treatment [75], suggesting that the effects of SVCT2 are tissue-specific. On the other hand, vitamin C can act as an electron donor for the reduction of mitochondrial cytochrome C [76]. Strikingly, the reduction of cytochrome C mediated by AA results in its oxidation (and eventual generation of DHA), which could generate oxidative stress. Thus, vitamin C could have both a “protective mode” and a “destructive mode” in mitochondria [77]. The “protective mode” would work in an environment with physiological doses of AA, where it would be constantly reduced without affecting the mitochondrial redox potential [77]. In high doses, the “destructive mode” could be activated where the reduction of cytochrome C in the mitochondrial intermembrane space at the expense of AA would favor its oxidation to DHA, which would alter the electron transport chain and NADH levels and would trigger a decrease in the coenzyme Q pool [77], which would result in cell death due to an overwhelming overload of mitochondrial stress.

## 7. Regulation of Cell Death by Vitamin C

Vitamin C’s role in cell death processes is controversial since antioxidants have historically been considered to have cytoprotective effects. Several studies support this hypothesis, specifically with AA as a molecule that prevents cell death under conditions of oxidative stress. For example, AA is capable of inhibiting H_2_O_2_-induced cell death in leukemia cells in a GSH-dependent manner [76], inhibiting genotoxic stress damage and mutations in DNA [77]. AA is also capable of preventing ischemic damage and inhibiting Caspase-8 activity in cardiac and cancer epithelial cells [35,78,79]. At the same time, other studies have reported that AA has an inhibitory effect on apoptosis, preventing neurodegeneration of the hippocampus in response to neurotoxins by decreasing the expression of Bax and increasing the levels of Bcl-2 [19,80], key proteins in the induction of intrinsic apoptosis (Figure 1B). Vitamin C may also inhibit TRAIL-dependent extrinsic apoptosis in monocytes and breast cancer lines, decreasing the activity of Caspase-3, -8, and -10 [34,35,81]. In addition, another study suggested that the combination of ascorbate 2-phosphate (AA-2P), an oxidation-resistant vitamin C analog, with N-acetylcysteine (NAC), would have synergistic effects in protecting against damage induced by H_2_O_2_ (500 µM) in human mesenchymal stem cells, preventing mitoptosis and mitochondrial fission through inhibition of a protein related to dynamin 1 (Drp1) [82]. Thus, this combination may protect cells from necroptosis and apoptosis [82]. Interestingly, this effect was observed with the lowest concentration of AA-2P as the AA-2P/NAC combination loses its synergistic capacity and acquires antagonistic properties against oxidative damage with increasing AA-2P [82]. This suggests that an eventual AA overload could favor its intracellular oxidation and subsequent generation of DHA, which could induce death independently of NAC antioxidant activity.

Tumor cells are characterized as having great reducing power and being capable of incorporating the oxidized form of vitamin C, DHA, through GLUTs, which can be reduced and accumulated intracellularly to AA at the expense of GSH or other electron donors [12,13,33,35,83]. This experimental condition, where only the protective effects of vitamin C are observed, can be attributed to the cell model and experimental conditions used, since there is abundant evidence that indicates that vitamin C can act as a pro-oxidant in other models. The lethal action of vitamin C on cancer cells is not recent; more than 40 years ago, it was postulated for the first time that treatment with pharmacological doses of vitamin C could promote tumor remission in patients [84]. More recently, in vitro studies have elucidated in detail the mechanism by which treatment with megadoses of AA would favor tumor death [31,32,85]. Specifically, it has been postulated that the megadoses of AA, in the presence of metals, favor the Fenton reaction and the subsequent extracellular production of H_2_O_2_ [7,31,32,86]. Diffusion of H_2_O_2_ from the extracellular medium could be the trigger for an overwhelming overload of intracellular ROS, which would favor tumor death due to conventional necrosis [31,86,87]. It is important to note that this mechanism only works with megadoses of AA, because during the diffusion of H_2_O_2_, it is diluted 100-fold [88]; therefore, high concentrations of AA are needed to produce a lethal amount of H_2_O_2_. It is important to highlight that AA antitumor activity has been tested in patients by administering megadoses of AA intravenously (IV) [7]. Thus, to date there are more than 15 clinical studies in progress where IV AA is administered in combination with other antineoplastics in order to find potentiating effects [7]. At the same time, it has been determined that the use of IV AA is completely safe in patients, even in combination with radiation and temozolomide, as a possible treatment against glioblastoma [89] or other types of cancer, such as pancreatic, non-small-cell lung cancer, and refractory lymphoma, among others [7].

Of note, the antitumor function of vitamin C through the extracellular production of H_2_O_2_ would be reserved exclusively for AA, and not for its nonoxidizable analog, AA-2P, or its oxidized form, DHA [83], because extracellular production of H_2_O_2_ requires the release of protons from AA [31,32]. However, neither AA-2P nor DHA releases protons in solution. Conversely, DHA needs to remove protons from the medium for its reduction. In this context, DHA would be unable to promote H_2_O_2_ production even with megadoses of this molecule [40]. At the same time, AA-2P is an excellent control when one wishes to evaluate the antitumor effects of AA independent of H_2_O_2_ production. 

Studies to date have shown that AA-2P is a transportable form of vitamin C exclusively dependent on SVCTs; esterases are required to remove phosphate groups, allowing its uptake independent of the production of H_2_O_2_ [83]. In line with this hypothesis, catalase treatment has been shown to inhibit the increase in ROS and cell death induced by megadoses of AA. Surprisingly, catalase has no protective effect against treatment with megadoses of DHA [40]. This evidence supports the hypothesis that DHA could induce tumor death by a mechanism independent of H_2_O_2_ production and, therefore, eventually independent of conventional necrosis. Interestingly, DHA antitumor effects can be exacerbated in cells that overexpress GLUT1 [89,90,91] that occurs as a result of hypoxic conditions observed in many tumors and is dependent on HIF1-α [92,93]. Thus, DHA may be a possible target for the treatment of tumors that overexpress GLUT1 or that are in hypoxic conditions and are resistant to megadoses of AA. In this sense, it is important to highlight that the use of pharmacological doses of AA in patients is safe and cheap [94,95]. Unfortunately, in a phase II clinical trial, up to 60 g of AA were injected intravenously in patients with castration-resistant prostate cancer with no effect on tumor remission [94]. This suggests that, eventually, AA alone could not induce tumor death in humans. Although the use of vitamin C in combination with other therapies has been suggested, special attention must be paid to the combined use of classical apoptosis death inducers, such as doxorubicin, since vitamin C can antagonize its effects in vivo [33]. Thus, the use of new nonapoptotic death-inducing compounds could be an interesting target to enhance the antitumor effects of vitamin C.

In a nontumor context, studies have evaluated whether DHA could be a pro-oxidant molecule with most carried out in brain tissue since this organ is the one with the highest accumulation of vitamin C in the body: up to 10 mM in neurons [3,6]. In rat brain sections, intracellular production of DHA or its uptake from the extracellular environment favors lipid peroxidation, consumption of GSH and the production of ROS [96,97,98]. At the same time, DHA is a potent disruptor of neuronal metabolism as isolated cortical neurons that are loaded with AA rapidly oxidize it, accumulating DHA [17]. In turn, DHA in the neuron increases the consumption of GSH, stops glycolysis and increases the pentose pathway [17]. These metabolic alterations generated by DHA may explain the death phenomena associated with vitamin C, where the intracellular production of DHA from AA, a product of oxidative stress, would be the possible trigger for cell death [19]. This hypothesis, where DHA could trigger neuronal death has been tested by our work group in neurons that have been stressed by H_2_O_2_ treatment and subsequent incubation with DHA [18], a condition that dramatically favors cell death. Interestingly, neuronal death triggered by DHA can be prevented by bystander cells that recycle DHA [13], such as astrocytes (Figure 1A). These cells that can capture DHA through GLUT1 [18], reduce it to AA and carry out efflux of this molecule [17], recycling vitamin C between astrocytes and neurons [3,12], which would favor cell survival. Vitamin C recycling between astrocytes and neurons has been less studied in pathophysiological contexts, and the implications of conditions characterized by astrocyte dysfunction and thus inefficient DHA recycling are unknown for the neuron (Figure 1A).

Accumulating evidence suggests that the induction of cell death regulated by megadoses of AA would correspond to conventional necrosis due to extracellular production of H_2_O_2_. On the other hand, DHA could induce death by a mechanism with characteristics of necrotic disintegration not yet determined.

## 8. Regulation of Necroptosis by Vitamin C: AA as Apoptosis Inhibitor and DHA as RIPK1 Activator

Necroptosis is a pathway that has recently been characterized, and its scope determined at a physiological and pathophysiological level. After determining that receptor-interacting serine/threonine-protein kinase 1 (RIPK1) regulated cell death due to necroptosis, three independent research groups reported and characterized another protein that was necessary for the execution of this pathway, RIPK3 [99,100,101]. Knockout animals and genome-wide siRNA screening, showed that RIPK3 triggers a metabolic alteration that changed the response to TNF from apoptosis to necroptosis [100,101] that required Caspase-8 (Casp8) to be inhibited or absent [102]. Furthermore, in response to TNF, an interaction occurred between RIPK1-RIPK3-Casp8 and the death adapters TNFR1-associated death domain protein (TRADD) and Fas-associated protein with death domain (FADD) [102]. This set of proteins (RIPK1/RIPK3/Casp8/TRADD/FADD) was called complex IIb, to differentiate it from the canonical activation pathway of apoptosis in response to TNF, composed of casp-8/FADD/TRADD, called complex IIa [103,104]. However, the existence of the necroptosis executor protein, mixed lineage kinase domain-like (MLKL), which is activated by RIPK3-dependent phosphorylation, was subsequently determined [105,106]. The discovery of MLKL showed that complex IIb was not necessary for the execution of necroptosis due to the existence of another complex called the “necrosome,” composed only of RIPK1/RIPK3/MLKL [103]. Activation of MLKL by phosphorylation induces formation of tetramers and their translocation to the plasma membrane [106,107], resulting in the exposure of phosphatidylserine and the formation of pores that trigger stress osmotic and death by cell explosion. Cell death by the MLKL-dependent necroptotic pathway induces the release of damage-associated molecular pattern (DAMP), which induces inflammation, greater activation of necroptosis, and eventually other parallel death pathways [108].

Current studies suggest that RIPK1 is the critical protein that regulates cell death mechanisms of apoptosis and necroptosis [109,110,111]. The regulation of RIPK1 depends mainly on two factors: ubiquitylation and phosphorylation [112,113,114]. The ubiquitination of RIPK1 depends on the activity of cIAP1/2 and LUBAC that act as E3 ubiquitin ligases [115]. With regard to the regulatory mechanisms of RIPK1 phosphorylation, these are only beginning to be elucidated. It is known that RIPK1 can also be inhibited through phosphorylation by IKKα/β [116]. The activity of IKKα/β on RIPK1 is independent of the activation of NF-κB. Under physiological conditions (absence of death stimuli), IKKα/β directly phosphorylates RIPK1 in an inhibitory manner, maintaining it in the plasma membrane and preventing the formation of complex IIa, IIb or the necrosome [116]. Alternatively, when IKKα/β complex is inhibited, an inhibitory hypophosphorylation of RIPK1 occurs, which induces the activation of RIPK1 mediated by the autophosphorylation of serine 166 and finally triggers necroptosis or uncontrolled apoptosis [116]. RIPK1 can also be inhibited by MAPK-activated protein kinase 2 (MK2) in the cytosol [117] by direct phosphorylation of serines 321 and 336, independent of IKKα/β activity [117,118]. MK2-mediated phosphorylation of RIPK1 prevents complex IIb and necrosome formation in the cytosol independent of death ligands (intrinsic control of necroptosis) because it prevents RIPK1 from being able to bind Casp-8 or FADD [118], thus preventing the activation of apoptosis or necroptosis (depending on the stimulus) (Figure 1B,C). The signaling pathways that regulate apoptosis and necroptosis are closely related. Generally, both routes act as negative feedback for the other. Thus, when death ligands, such as TNF or FasL, bind their receptor, they activate complex IIa, activating Casp-8 that cleaves RIPK1 at aspartate 324, favoring apoptosis [119]. Conversely, when there are death stimuli and pro-apoptotic proteins, such as Casp-8 and BAX, are inhibited or expressed at low levels and/or antiapoptotic proteins, such as those of the Bcl-2 family, are overexpressed, necroptosis is activated via complex IIb or the necrosome [120]. 

Interestingly, the reduced form of vitamin C, AA, has been widely reported as an inhibitor of apoptosis because it decreases the activity of Casp-3 and Casp-8, increases the levels of Bcl-2, prevents the release of cytochrome C from the mitochondria, and decreases the expression of BAX [19,34,35,78,79,80]. Thus, under physiological conditions, AA would be a potent inhibitor of apoptosis (Figure 1B). Conversely, vitamin C can induce death in tumor cells, largely due to necrosis [31,32,90,121]. However, current literature indicates that there is a signal transduction mechanism specifically regulated by the oxidized form of vitamin C, DHA. The first studies that postulated vitamin C as a regulator of signal transduction pathways were activating NF-κB with TNF and determined that vitamin C was a potent inhibitor of this pathway [20,21]. Subsequently, DHA inhibition of NF-κB activity via inhibiting the kinase activity of IKKα/β and p38 in response to TNF was shown [22,27]. In this scenario, DHA would be a potent inhibitor of survival signals by suppressing the activation of NF-κB (Figure 1C). As a consequence of the production and/or accumulation of DHA in the cell, cell death is finally triggered [18,89]. In line with this notion, and as described above, there is a close relationship between the effects of DHA and the regulation of necroptosis through RIPK1. Under physiological conditions, vitamin C is found as AA; therefore, IKKα/β is active, keeping RIPK1 phosphorylated in an inhibitory way [116]. At the same time, under physiological conditions, p38 activates MK2 by phosphorylation, which inhibits RIPK1 phosphorylation in the cytoplasm [117,118]. Interestingly, DHA specifically inhibits IKKα/β and p38 [22,27]. 

Accumulating evidence suggests that DHA primarily targets the activation of RIPK1 to prevent its inhibitory phosphorylation. At the same time, AA would precondition cells to necroptosis as it is a potent inhibitor of apoptosis [34,35]. Thus, under pathophysiological conditions where the oxidation of AA to DHA is favored, DHA could stimulate the induction of cell death mainly by necroptosis (Figure 1B,C). To meet this requirement, it is necessary for cells to accumulate a sufficient amount of vitamin C to trigger intracellular death stimuli. Thus, the pro-necroptotic effect of DHA could be limited only to cells that accumulate high concentrations of vitamin C, which have a limited capacity to keep AA reduced, are highly sensitive to oxidative stress, and are also refractory to apoptosis. Unfortunately, the only cells that meet these requirements are neurons, which accumulate up to 10 mM AA intracellularly [6], rapidly oxidize AA to DHA [17], and, in the adult brain, are refractory to apoptosis, expressing very low levels of Casp-8 and -3 and overexpressing antiapoptotic proteins [122,123]. 

## 9. Conclusions

To understand the complexity of vitamin C, several studies have been carried out in normal and tumor cells, with contradictory results. On the one hand, there is the classic current, which points out that vitamin C has a protective effect in various pathophysiological conditions and oxidative stress [35,79,81,82]. Conversely, reports of the pro-oxidant properties of vitamin C in a tumor or nontumor context are emerging [3,48,50,90,96,97,98,121,124]. The current problem lies in the fact that most studies point out that the observed results are produced by “vitamin C,” but it is not specified whether it is due to its reduced (AA) or oxidized (DHA) form. In addition, the extent of the reducing capacity of each cell line or model to maintain vitamin C in the form of AA is not clear, nor is it stated whether the conditions permit vitamin C recycling by bystander cells [12,18]. Furthermore, it has been suggested that DHA could be pharmacologically active and capable of killing tumor cells [89]. The mechanisms by which DHA could induce cell death are still poorly understood, but it could alter glucose metabolism, causing an energy imbalance [17,89], which would converge in some type of cell death that has not yet been characterized. We postulate that, to resolve the contradictory effects attributed to vitamin C, it is necessary to understand the function of DHA, the pathways regulated by this molecule, and the effects of its chronic accumulation due to the absence of recycling or deficient recycling.

## Figures and Tables

**Table 1:** Molecular pathways regulated by vitamin C in different cell models.

Cell Type	Treatment	Effect	Ref.
ECV304, HUEVEC, HeLa.	AA/DHA	Inhibition translocation of NF-kB, inhibition phosphorylation of IkB	[20,21,22]
MCF7, HL-60, HUVEC, HeLa.	DHA	Inhibition phosphorylation of IKKα/β and p38	[22,27]
HL-60, NB4, NB4-R1, Neuro2a, HUVEC.	AA	Induction of ERK phosphorylation	[10,30]
B16F10, HL-60.	AA/DHA	Activation of p38; suppression of p42/44	[28,37,39]
MDA-MB-231.	AA	Arrest S-phase	[40]
AS52 C8D1A.	AA/DHA	Arrest G_2_/M	[41,42]
Neuro2a, HT-1080.	AA	RIPK1 overexpression	[19,43]
